# Development of a Nutrient Profiling Model for Processed Foods in Japan

**DOI:** 10.3390/nu16173026

**Published:** 2024-09-07

**Authors:** Jun Takebayashi, Hidemi Takimoto, Chika Okada, Yuko Tousen, Yoshiko Ishimi

**Affiliations:** 1 National Institute of Health and Nutrition, National Institutes of Biomedical Innovation, Health and Nutrition, 3-17 Senrioka Shinmachi, Settsu-shi, Osaka 566-0002, Japan; jtake@nibiohn.go.jp (J.T.); c-okada@nibiohn.go.jp (C.O.); tousen@nibiohn.go.jp (Y.T.); 2 Tokyo NODAI Research Institute, Tokyo University of Agriculture, 1-1-1 Sakuragaoka, Setagaya-ku, Tokyo 156-8502, Japan; yi207200@nodai.ac.jp

**Keywords:** scoring algorithm, rating algorithm, across-the-board criteria, Japanese food culture

## Abstract

Numerous nutrient profiling models (NPMs) exist worldwide, but Japan lacks an official NPM. Using the Australian and New Zealand Health Star Rating (HSR) as a reference, “Processed Foods in Japan version 1.0” (NPM-PFJ (1.0)) was developed to fit Japanese food culture and policies. In total, 668 processed foods from the Standard Tables of Food Composition in Japan were analyzed, excluding seasonings/spices, fats/oils, alcoholic beverages, and infant food. The NPM-PFJ (1.0) scoring algorithm was adapted from HSR, with revised reference values for energy, saturated fat, total sugars, sodium, protein, and dietary fiber in alignment with Japanese standards. Reference values for fruits, vegetables, nuts, and legumes (fvnl) remained unchanged. Median scores were 4.5 for HSR and 5.0 for NPM-PFJ (1.0), showing high correlation (r = 0.939, *p* < 0.01). Thereafter, food categories familiar and meaningful in Japan were defined based on a hierarchical cluster analysis of scoring patterns, creating six categories with distinct characteristics. Finally, the rating algorithm for NPM-PFJ (1.0) was created using each group’s score distribution (10th percentile). The NPM-PFJ (1.0) was developed through a fully transparent and evidence-based process and is expected to facilitate the reformulation of food products by food industries and help consumers easily access healthier processed foods. This model marks a significant step forward in developing an NPM tailored to Japanese food culture and health policies, with the potential to enhance public health.

## 1. Introduction

Nutrient profiling is “the science of classifying or ranking foods according to their nutritional composition for reasons related to preventing disease and promoting health” [1] and serves diverse purposes [2,3,4,5]. For example, it can inform consumers about healthier food choices, guide food industries in reformulating products to be healthier, establish guidelines for school lunches, regulate health claims on food packaging, and restrict food advertising and marketing. A nutrient profiling model (NPM) is a practical tool for assessing the nutrient content of foods rather than diets [6,7]. Numerous NPMs have been developed worldwide [2,3,5,8,9,10], each with a unique purpose [11]. NPMs are roughly divided into two classes: “across-the-board” or “category-specific” criteria [6,11,12,13,14].

Across-the-board NPMs use a single or limited set of nutrient criteria to allocate “scores” based on the contents of multiple nutrients to rank foods: they help consumers select healthier foods across categories, but may be challenging for food industries producing foods intrinsically high in fat, sugar, and salt. Representative across-the-board NPMs include the Nutri-Score and the Health Star Rating (HSR) system. Nutri-Score, adopted in France in 2017, is characterized by its five-tier color-coded front-of-pack nutrition labeling system ranging from ‘A’ (healthiest) to ‘E’ (least healthy) [15]. It focuses on reducing the intake of energy, sugars, saturated fats, and sodium while promoting the consumption of fruits and vegetables, fiber, and protein. Nutri-Score has been adopted by several European countries, including Belgium, Switzerland, Germany, Luxembourg, The Netherlands, and Spain [16]. Barrett et al. [17] indicated that Nutri-Score is related to diet-related disease risk and risk markers by the systematic review and meta-analysis. The HSR system, which is aimed at voluntary front-of-pack nutrition labeling, was adopted in Australia and New Zealand in 2014 [18]. The uptake of the front-of-pack nutrition label based on the HSR system in Australia increased to 40.7% of eligible products in 2019 [19]. Thomas et al. [20] indicated that the introduction of the HSR system encourages consumers to purchase healthier food items, based on household panel data in Australia. The HSR system has also been used to assess healthiness of foods in other countries worldwide [21,22].

Category-specific NPMs treat different categories of foods separately: they identify healthier options within each category without excluding entire categories, but may not be clear enough for consumers to switch from confectionery to fruit. Representative category-specific NPMs include the Keyhole and the World Health Organization (WHO) NPMs for Europe or South East Asia regions. The Keyhole is the longest-standing front-of-pack nutrition labeling system used in Sweden, Denmark, Norway, Iceland, Lithuania, and North Macedonia [10]. It aims to reduce the amount of one or more of the following: total fat, saturated and trans fatty acids, free sugars, salt (sodium), and/or increase amounts of fiber, whole grains, fruits, and vegetable. Wanselius et al. [23] investigated the impact of replacing foods in the dietary survey data of Swedish adolescents to those compliant with the Keyhole and observed reductions in the intakes of total fat, saturated fatty acids, monounsaturated fatty acids and free sugars. The WHO NPMs for Europe [24] or South East Asia regions [25] were developed to restrict the marketing or advertising of ‘unhealthy’ foods high in energy, saturated fats, sugars (total and added), non-sugar sweeteners, or sodium to children. These NPMs have been used to assess the healthiness of foods in specific countries [26,27].

However, Japan lacks an official NPM. An official NPM could assist the food industries to provide healthier food options, as well as increase the transparency of their product portfolios using unbiased indicators. The WHO emphasizes the importance of developing NPMs tailored to country-specific health issues and food cultures [5]. Therefore, a draft version of an NPM for processed foods in Japan (draft NPM-PFJ (thresholds)) was developed based on the mandatory nutrition labeling of processed foods [28]. This draft NPM-PFJ (thresholds) aimed to support front-of-pack labeling and encourage voluntary reformulation by food industries towards healthier options. It employed a category-specific model with thresholds to classify foods with high levels of nutrients that should be restricted, such as total fat, saturated fat, sodium (salt equivalent), and energy, based on “per 100 g (mL) of food”. Seasonings/spices and fats/oils were excluded, as they are consumed as ingredients in Japanese dishes rather than as standalone foods [29].

The challenge with this draft version was its limited ability to evaluate the overall healthiness of processed foods. This draft version did not enable direct comparisons beyond categories or distinguish between foods high in multiple restricted nutrients and those high in only one restricted nutrient. Hence, this study aimed to develop a more comprehensive NPM, employing an across-the-board model with scoring algorithms to objectively measure the healthiness of food products while fitting Japanese food culture and policies.

This novel NPM, designated as the NPM for processed foods in Japan version 1.0 (NPM-PFJ (1.0)), could be one of the candidates for an official NPM in Japan. NPM-PFJ (1.0) was developed on the basis of objective nutrition science, in accordance with the development concept detailed in the Materials and Methods section. Existing NPMs worldwide were reviewed, and the HSR system was found to align the best with the concepts, although further adaptation to Japanese food culture and policies was needed. Consequently, using the HSR system as a reference, NPM-PFJ (1.0) was fundamentally developed. The research questions addressed in this study are: (1) How well does the HSR system align with Japanese food culture and policies? (2) What adaptations are necessary to improve its applicability in Japan? Hence, NPM-PFJ (1.0) would provide a more accurate and holistic evaluation of the nutritional quality of processed foods in Japan, promoting healthier food choices and contributing to public health.

## 2. Materials and Methods

### 2.1. Development Concept of NPM-PFJ (1.0)

(1) The target group for NPM-PFJ (1.0) was individuals aged 18 years or older in Japan. (2) Nutrients to be included, considering their public-health importance in Japan, were as follows: energy, protein, and sodium (mandatory nutrition labeling [30]); saturated fat and dietary fiber (recommended nutrition labeling [30]); vegetables (recommended by the national health promotion plan, the Health Japan 21 (third term) [31]); and total sugars (guideline of the WHO [32]). (3) Seasonings/spices, fats/oils, ready-to-eat meals (such as boxed meals and delicatessen foods), alcoholic beverages, and infant food were excluded. Seasonings/spices, fats/oils, and ready-to-eat meals were handled in the NPM for dishes in Japan, as described in a separate study [33]. (4) The scoring algorithm should be explicitly and logically linked to public health recommendations in Japan. (5) Generated scores were assessed to rank foods with a small number of categories to ensure feasibility for food industries. Food categorization should be based on scientifically sound and objective methods and was adapted to fit Japanese food culture. (6) Reference amounts/units were described per 100 g or 100 mL because Japan has no publicly standardized food-serving sizes. (7) The NPM development process is completely transparent and based on open-source data. The entire process to develop the algorithms is presented here, including numerical evidence and the complete data set.

### 2.2. Selection of the Reference NPM

Previously published reviews by Martin et al. [2] and Labonté et al. [3] were used to identify a suitable reference NPM. These reviews focused on NPMs used in government-led nutrition policies and summarized their key characteristics. For applicability in Japan, the following selection criteria for the reference NPM were established:

1. Specific nutrients and food components “to limit” or “to encourage” are included in the Japanese nutrient labeling system.
2. The total number of food categories, including nutrient criteria (major, sub-, and sub-subcategories combined), is less than those in the Standard Tables of Food Composition in Japan (eighth revised edition) (STFCJ-8) [34] (*n* = 18).
3. Number and type of nutrients and food components are consistent across the model’s food categories and types of food products evaluated.
4. The model covers foods for adults.
5. Reference amounts/units are per 100 g or 100 mL.

A total of 229 models were examined for eligibility as the reference NPM model. Eighteen models met the criteria. Considering the development concept of NPM-PFJ (1.0), the Health Star Rating (HSR) System [18] was selected as the reference model. The fundamental concepts of the HSR are presented in Table 1.

### 2.3. Food Composition Data

To ensure the transparency of NPM-PFJ (1.0), food composition data need to be reliable and publicly accessible [38]. Thus, the composition of the 668 processed foods, the same as those previously used in the development of the draft NPM-PFJ (thresholds) [28], was obtained from STFCJ-8 [34]. Seasonings/spices, fats/oils, alcoholic beverages, and infant foods were excluded. Seasonings/spices and fats/oils were excluded because they were handled in the NPM for dishes in Japan [33]. Alcoholic beverages were excluded as they are not typically included in NPMs due to their distinct consumption patterns and health impacts. Infant foods were excluded because the target group for NPM-PFJ (1.0) was individuals aged 18 years or older. 

In STFCJ-8, the FAO/INFOODS method [39] was applied for energy calculations. There are two values for proteins (one calculated as the sum of amino acid residues and the other from the reference nitrogen) and multiple values for fat, carbohydrates, and dietary fiber owing to updates in analytical methods. However, these updated values differ conceptually from those used in Japanese nutrient declarations. Therefore, the values for energy, available carbohydrates, and total sugars were calculated individually according to previously reported methodologies [40]. Table S1 provides all food composition data, including imputation methods.

### 2.4. Scoring Algorithm for the NPM-PFJ (1.0)

**Figure 1:**
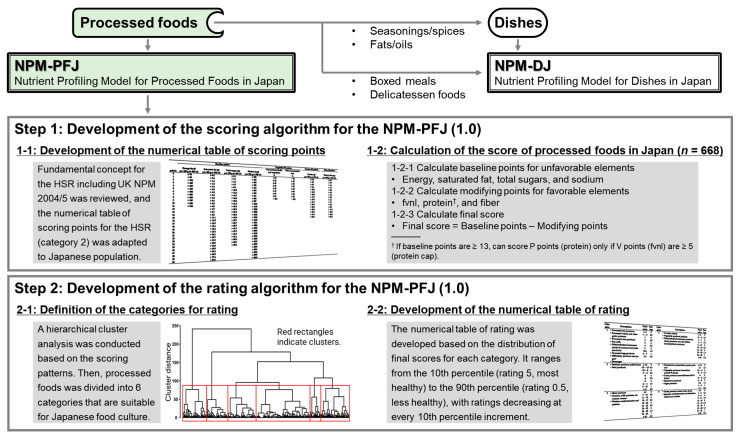
Development scheme for the NPM-PFJ (1.0).

The development scheme for the NPM-PFJ (1.0) is shown in Figure 1. The scoring algorithm was created by adapting the fundamental concepts of the HSR [18,37,41] (Table 1) and UK NPM 2004/5 [35,36,42], which serves as the basis for the HSR, to fit the Japanese population (Table 2). The reference values for energy, saturated fat, total sugars, sodium, protein, and dietary fiber were revised in alignment with Japanese standards [30,31,32,43], whereas the values for fvnl (fruits, vegetables, nuts, and legumes, including coconut, herbs, fungi, seeds, and algae) remained unchanged. The extension methods followed the HSR approach in linearly extended regions and were adjusted to HSR values using the weighted average (Table 3). Detailed calculations for the numerical tables of points for NPM-PFJ (1.0) are shown in Table S2. The protein cap (products scoring ≥13 HSR baseline points cannot score points for protein unless they score five or more HSR V points) was also employed, with baseline points and final scores calculated similarly as follows.

Baseline points = energy points + saturated fat points + total sugar points + sodium points(1)

Final score = baseline points − V points − P points − F points(2)

### 2.5. Creating the Rating Algorithm for the NPM-PFJ (1.0)

Following the proposed scoring algorithm in Table 3, points for energy, saturated fat, total sugars, sodium, fvnl, protein, and dietary fiber were calculated for the 668 processed foods. Hierarchical cluster analysis using Ward’s method was applied to create food groups, and the distribution of scores (10th percentile) was calculated for each group.

### 2.6. Statistical Analysis

Basic statistical analyses were performed using Microsoft Excel 2021 (Microsoft Corporation, Redmond, WA, USA), whereas R version 4.4.1 (R Foundation for Statistical Computing, Vienna, Austria) was used for data visualization (‘boxplot’ function in the R package ‘graphics’ (version 4.4.1)), Pearson correlation analysis (‘cor.test’ function in the R package ‘stats’ (version 4.4.1)), and cluster analysis (‘hclust’ function in the R package ‘stats’ (version 4.4.1)).

## 3. Results

### 3.1. Comparisons between HSR Scores and NPM-PFJ (1.0) Scores

**Figure 2:**
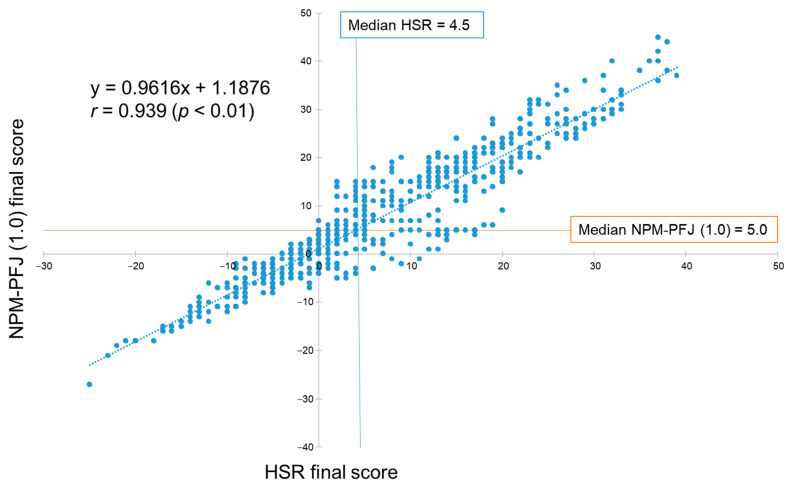
NPM-PFJ (1.0) final score vs. HSR final score (*n* = 668). Each blue dot represents an individual data point. The blue dashed line represents the regression line. The Pearson’s correlation coefficient (r) is shown.

The 668 processed foods were assessed using the HSR and the NPM-PFJ (1.0) models. The final scores were plotted and compared (Figure 2). Median scores were 4.5 for HSR and 5.0 for NPM-PFJ (1.0). The correlation between these scores was high (r = 0.939, *p* < 0.01). The results for all processed food scores in Japan using NPM-PFJ (1.0) and HSR are detailed in Table S1.

### 3.2. Development of Food Groups for NPM-PFJ (1.0)

Hierarchical clustering using Ward’s method divided the data into six clusters. The cluster assignments for each food product are presented in Table S1. Some foods were classified into different clusters but perceived as similar by consumers and thus subject to comparison. To address this issue, these items were regrouped based on the criterion of unifying them into the cluster with the highest frequency of similar foods. For example, rye bread (Cluster 2) and bread rolls and croissants (Cluster 3) were grouped into Category 1, as most other breads were in Cluster 1. The final food categories of all food products, with individually annotated regrouped items, are shown in Table S1 under “Food Categories of NPM-PFJ (1.0)”.

**Figure 3:**
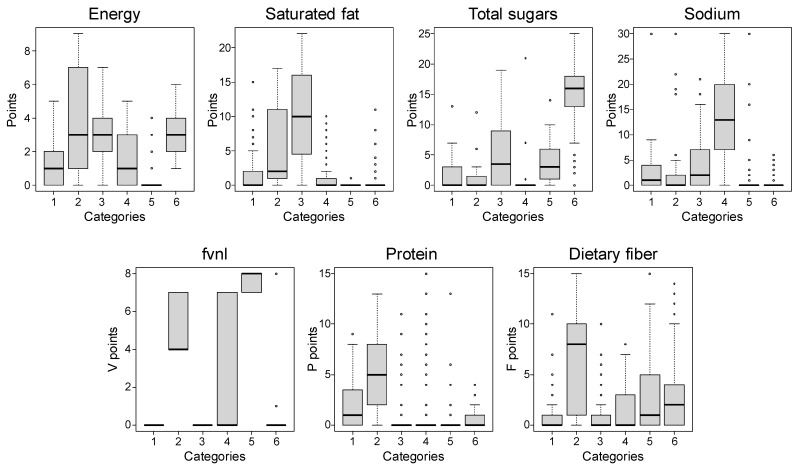
Characteristics of the scoring pattern in each category of the NPM-PFJ (1.0). The n number of each category is as follows: category 1 = 148; category 2 = 57; category 3 = 126; category 4 = 157; category 5 = 74; and category 6 = 106. The box in the plot represents the interquartile range (IQR), which is the range between the first quartile (Q1) and the third quartile (Q3). The line inside the box marks the median. The whiskers extend from the box to the minimum and maximum data values, excluding any outliers. In the presence of outliers, the whiskers extend to a maximum of 1.5 times the IQR from the box. Outliers are data points that fall below Q1 − 1.5 × IQR or above Q3 + 1.5 × IQR. These are represented by dots in the plot.

The distribution of foods according to the HSR and NPM-PFJ (1.0) categories is shown in Table 4. Most Japanese foods (*n* = 565, 85%) were classified as “2. Foods” according to HSR. The number of foods in each category for NPM-PFJ (1.0) was higher than for HSR. The distribution of each score and the final NPM-PFJ (1.0) score by food category is shown in Figure 3. Each food category had distinct scores: Category 2 foods had the highest median scores for energy, protein, and dietary fiber; Category 3 foods for saturated fat; Category 4 foods for sodium; Category 5 foods for fvnl; and Category 6 foods for total sugars. 

### 3.3. Development of Ratings for Each Food Group for NPM-PFJ (1.0)

Table 5 presents a numerical table of ratings for NPM-PFJ (1.0). The highest rating of 5 (the healthiest) corresponds to the 90th percentile or lower of the final score in each category (Table S3). For each decrease of 0.5 in the rating, the upper limit was set to the next 10th percentile of the final score as an integer. The lowest rating of 0.5 (the least healthy) corresponds to the 10th percentile or higher of the final score in each category. Category 1 included a variety of foods; Category 2 consisted of soybean products (solid form) and seed products; Category 3 included meat, dairy products, Western confectioneries, and pastries; Category 4 contained high-sodium foods such as pickles and dried/salted fishes; Category 5 included most plant-based foods; and Category 6 comprised fruit products and Japanese confectioneries.

The ratings for food items within each category were approximately as follows:

- Category 1: Beverages (teas) ≈ noodles (uncooked/boiled) ≈ soy milk ≥ processed rice products ≈ fish/mollusk/crustacean products (canned) ≈ ≥ yogurt ≥ milk/dairy products ≈ breads ≥beverages (others) ≈ fish/mollusk/crustacean products (paste) ≈ processed egg products ≈ processed corn products. Overall, the high ratings in this category were mainly due to the low amounts of negative nutrients. Individually, in fish/mollusk/crustacean products (canned), the relatively high amounts of saturated fat and sodium were offset by the relatively high amounts of protein. In breads and processed corn products, the amount of dietary fiber (1.2–10.5 g/100 g, Table S1) also contributed to their ratings.
- Category 2: Soybean products (solid-form) ≥ seed products. Seed products have a relatively higher protein and dietary fiber content compared to soybean products, but they contain even more saturated fat.
- Category 3: Ice creams ≥ pastries ≥ Western confectioneries (unbaked) ≥ meat products ≥ Western confectioneries (baked) ≈ cheeses, milk powders, and creams. For meat products, the sodium content also contributed to their ratings. For baked Western confectioneries, the total sugar content was a significant factor in their ratings.
- Category 4: Vegetable products (pickles) ≥ noodles (dried) ≥ fish/mollusk/crustacean products (dried products and salted/simmered/pickled products). Most items in this category have uniformly high sodium levels. Thus, the ratings were considerably influenced not only by the sodium contents, but also by other nutrients such as fvnl, protein, and dietary fiber.
- Category 5: Vegetables products (canned/frozen) ≥ potato/other potato products ≥ algae products ≥ vegetable juices (100%) ≥ fruits juices (100%) ≥ mushrooms products ≥ processed fruits (canned/frozen). All products in this category primarily consist of fvnl, resulting in similar V points. Therefore, the contents of total sugars and sodium determined most of the rating.
- Category 6: Dried fruits ≥ Japanese confectioneries ≥ jams ≥ candies. For dried fruits, high ratings were largely attributed to their fvnl and dietary fiber content.

## 4. Discussion

In this study, we developed a novel NPM, NPM-PFJ (1.0), to assess Japanese processed food products. The HSR was chosen as the reference model for three key reasons. (1) Nutritional perspective: the nutrients and food components considered in the HSR closely align with the concept of NPM-PFJ (1.0). (2) Practical perspective: The HSR employs algorithms that can be effectively applied to NPM-PFJ (1.0). Specifically, the HSR generates scores using nutritional values per 100 g or 100 mL and rates food healthiness by six separate categories. (3) Methodological perspective: the numerical basis of the HSR scoring algorithms (including UK NPM 2004/5) is traceable and can be adapted to fit public-health recommendations in Japan.

The NPM-PFJ (1.0) scores showed a strong correlation with HSR scores (Figure 2), suggesting that the scoring algorithm for NPM-PFJ (1.0) provides a comparable evaluation of Japanese food products. This comparability would assure food industries when disclosing their product portfolios using NPM-PFJ (1.0). However, the food categories in the HSR do not align with Japanese food culture, as three of the six categories are dairy products (Table 4). Defining food categories suitable for each culture or cuisine is crucial for national processes [5]. The food categories for NPM-PFJ (1.0) were determined scientifically, using a hierarchical cluster analysis with Ward’s method based on the scoring patterns of processed foods in Japan (Figure 1 and Figure 3). Thus, compared to HSR categories, categories for NPM-PFJ (1.0) (Table 5) are more familiar and meaningful to Japanese consumers and food industries. For instance, most foods in Category 4 are often deemed “unhealthy” owing to their high sodium content. Despite ongoing efforts by the government, industry, and academia to reduce the population’s sodium intake, the 2019 National Health and Nutrition Survey in Japan [44] reported a mean sodium intake of 3828 mg/day (approximately 3.8 g/day) among Japanese people aged 1 year and over, which is nearly double the WHO recommendation of less than 2 g/day [45], indicating the difficulty of reducing the sodium intake in Japan. The novel rating algorithm for NPM-PFJ (1.0) categorizes sodium-rich foods separately and evaluates their healthiness individually, which could help consumers choose healthier foods by motivating food companies to realistically reduce the sodium content in their products.

As described in the Results section, Category 1 includes a wide variety of foods, which may be confusing for consumers as well as food industries. To clarify the validity of the classifications of NPM-PFJ (1.0), further practical research is required. Reconsidering food categories and rating algorithms by applying nutrient data of food products currently sold could improve the alignment with Japanese food culture and dietary habits.

The strengths of NPM-PFJ (1.0) are as follows. (1) The scoring algorithm is explicitly and logically linked to public-health recommendations in Japan (Table 2 and Table S2). This linkage is crucial for the acceptance of NPM-PFJ (1.0) by the government and food industries. Additionally, a clear relationship with reference values allows for systematic updates in the future, similar to the UK NPM [42] and Nutri-Score [16]. (2) The rating algorithm is applied after being divided into several food categories suitable for Japanese food culture and environment. This approach may enable consumers to compare different types of foods to some extent, while ensuring feasibility for food industries. Notably, the HSR [18] and Nutri-Score [16] both employ “across-the-board” criteria, but aim to facilitate the comparison of similar types of foods only. In contrast, the Food Compass [46] aims to compare all foods in the same manner, which may pose challenges for food industries that mainly produce foods intrinsically high in nutrients to be restricted. (3) NPM-PFJ (1.0) was developed through a fully transparent and evidence-based process, remaining independent from external stakeholders. (4) NPM-PFJ (1.0) is expected to provide a comprehensive evaluation of the nutritional quality of processed foods distributed in Japan, promoting healthier food choices and contributing to public health. Several NPMs have been developed by food industries, both global [47,48] and Japanese [49,50], with the purpose of reformulating their own products. In contrast, NPM-PFJ (1.0) aims to enhance the overall quality of processed foods in Japan.

In Japan, the Strategic Initiative for a Healthy and Sustainable Food Environment, a comprehensive strategy involving multiple sectors and stakeholders, was launched in 2022 [51]. This initiative aims to reduce the excess sodium intake and address other nutritional and environmental challenges by collaborating with the government, industry, academia, and other stakeholders. NPM-PFJ (1.0) is tailored to the Japanese context and considers feasibility for a wide range of food industries without limiting to specific manufacturers. Moreover, NPM-PFJ (1.0) maintains high neutrality and transparency. Thus, NPM-PFJ (1.0) would be a strong candidate for an official NPM to support this initiative.

Several limitations were identified in this study: (1) The absence of certain data in the STFCJ, such as saturated fat, sugars, dietary fibers, and the proportion of ingredients comprising fvnl, may have reduced the score accuracy. An original method was employed to identify the non-condensed (%) fvnl in individual foods (Table S4). A methodology has been reported for estimating fvnl points from ingredient lists for processed foods in Canada [52]. Investigating the applicability of this method to Japan could be promising. (2) Some foods, like dried noodles, are evaluated under different conditions than when consumed, potentially leading to inaccurate scoring. Using post-cooked values could increase the accuracy. (3) The score distribution of the foods in this study may not fully reflect the actual market, as the STFCJ employs a single-component value per food item [34]. Further data collection on food products sold in Japan is necessary. For example, the Choices 5-Level Criteria [53] use large international product-specific food composition databases to create models. (4) The distribution of scores was particularly narrow for categories 1 and 5, preventing successful rating into 10 levels. These issues mentioned above may be resolved with an expanded database of food products sold in Japan.

In this study, traditional seasonings high in sodium, such as soy sauce and fermented soybean paste, were excluded. These seasonings are frequently used to flavor Japanese dishes. According to a previous study [54], the proportion of discretionary sodium intake among Japanese adults was 52.3% in men and 57.1% in women. This indicates the need for a more holistic approach to improving the healthfulness of Japanese foods, suggesting that a dish-based NPM, NPM-DJ, may be more appropriate for the Japanese diet [33].

## 5. Conclusions

The NPM-PFJ (1.0), a novel NPM employing an across-the-board model with scoring algorithms, was developed using the HSR as the reference model. The scoring algorithm for NPM-PFJ (1.0) is explicitly and logically linked to public-health recommendations in Japan, maintaining comparability to HSR. In contrast, the rating algorithm for NPM-PFJ (1.0) is based on uniquely defined food categories that are familiar and meaningful to the Japanese food culture and environment. Overall, NPM-PFJ (1.0) may enable consumers to compare different types of foods to some extent, while ensuring feasibility for food industries. The development process of NPM-PFJ (1.0) is fully transparent and evidence-based, remaining independent from external stakeholders. The novel NPM-PFJ (1.0) is expected to be valuable for food industries looking to disclose their product portfolios to consumers and investors. This model is anticipated to serve as a tool for the further reformulation of food products, facilitating easier access to healthier foods for consumers. This study’s limitations include missing data and narrow score distributions. An expanded database of food products sold in Japan may address these issues.

## Figures and Tables

**Table 1:** Fundamental concept for the HSR including UK NPM 2004/5.

Items	HSR (Including UK NPM 2004/5)	Reference
**Scoring algorithms**		
*Nutrients/food components*	Negative nutrients/food components (energy, saturated fat, total sugars, and sodium) and beneficial nutrients/food components (fvnl, protein, and dietary fiber).	
*Unit*	per 100 g or 100 mL (except for fvnl, %)	
*Score bands starting*		
• Energy	3.75% of **2130 kcal** (weighted average of DRVs for children aged 11–18 years (UK, 1991))	[35]
• Saturated fat	**11%** of food energy (DRVs, UK, 1991)	[35]
• Total sugars	**21%** of food energy (DRVs, UK, 1991)	[35]
• Sodium	3.75% of **2400 mg** (GDA for everyone over the age of 11 years (SACN, 2004))	[36]
• fvnl	25% of a total amount of a product (concentrated fruits or vegetables) or 40% of a total amount of a product (non-concentrated fvnl)	
• Protein	3.75% of **42 g** (weighted average of RNI for children aged 11–18 years (UK, 1991))	[35]
• Dietary fiber	3.75% of **24 g** (DRVs, UK, 1991)	[35]
*Methods of extension*		
• Energy	Extended linearly (2–11 points)	
• Saturated fat	Extended linearly (2–10 points) and extended non-linearly (11–30 points)	
• Total sugars	Extended linearly (2–25 points)	
• Sodium	Extended linearly (2–30 points)	
• fvnl	Extended empirically to 8 points	
• Protein	Extended linearly (2–5 points) and extended non-linearly (6–15 points)	
• Dietary fiber	Extended linearly (2–5 points) and extended non-linearly (6–15 points)	
*Protein cap*	If baseline points are ≥13, it can score P points only if V points are ≥5	
*Calculation*	Baseline points = energy points + saturated fat points + total sugar points + sodium pointsFinal score = baseline points − V points − P points − F points	
**Rating algorithms**		
*Categories*	**1. Non-dairy beverages, jellies, and water-based ice confections** **1D. Milk and Dairy beverages (and alternatives)** **2. Foods** **2D. Dairy foods (and alternatives)** **3. Oils and Spreads** **3D Cheese**	
*Methods of rating*	Score distribution **in a database of Australian foods**	[37]

Differences between the HSR and the NPM-PFJ (1.0) are highlighted in the bold and underlined text. DRV: dietary reference value, GDA: guideline daily amount, RNI: reference nutrient intake, SACN: Scientific Advisory Committee on Nutrition, UK NPM 2004/5: The United Kingdom nutrient profiling model developed by the food standards agency in 2004–2005.

**Table 2:** Fundamental concept for the development of NPM-PFJ (1.0).

Items	NPM-PFJ (1.0)	Reference
**Scoring algorithms**		
*Nutrients/food components*	Negative nutrients/food components (energy, saturated fat, total sugars, and sodium) and beneficial nutrients/food components (fvnl, protein, and dietary fiber)	
*Unit*	per 100 g or 100 mL (except for fvnl, %)	
*Score bands starting*		
Energy	3.75% of **2200 kcal** (NRVs (Japan, 2015))	[30]
Saturated fat	**7%** of food energy (DRIs, Japan, 2020)	[43]
Total sugars	**10%** of food energy (recommendation, WHO, 2015)	[32]
Sodium	3.75% of **2756 mg** (7 g NaCl, the Health Japan 21 (third term), 2023)	[31]
fvnl	25% of a total amount of a product (concentrated fruits or vegetables) or 40% of a total amount of a product (non-concentrated fvnl)	
Protein	3.75% of **81 g** (NRVs (Japan, 2015))	[30]
Dietary fiber	3.75% of **19 g** (NRVs (Japan, 2015))	[30]
*Methods of extension*		
Energy	Extended linearly (2–11 points)	
Saturated fat	Extended linearly (2–10 points) and **adjusted (11–30 points, weighted average with the HSR value)**	
Total sugars	Extended linearly (2–10 points) and **adjusted (11–25 points, weighted average with the HSR value)**	
Sodium	Extended linearly (2–30 points)	
fvnl	Extended empirically to 8 points	
Protein	**Adjusted (2–15 points, weighted average with the HSR value)**	
Dietary fiber	Extended linearly (1–5 points) and **adjusted (6–15 points, weighted average with the HSR value)**	
*Protein cap*	If baseline points are ≥13, it can score P points only if V points are ≥5	
*Calculation*	Baseline points = energy points + saturated fat points + total sugar points + sodium pointsFinal score = baseline points − V points − P points − F points	
**Rating algorithms**		
*Categories*	**Selection by cluster analysis**	
*Methods of rating*	Score distribution **(10th percentiles) of processed foods in the Standard Tables of Food Composition in Japan**	

Differences between the HSR and the NPM-PFJ (1.0) are highlighted in the bold and underlined text. DRI: dietary reference intake, NRV: nutrient reference value.

**Table 3:** Numerical table of scoring points for the NPM-PFJ (1.0).

	Baseline Points				Vegetable (V) Points		Protein (P) Points	Fiber (F) Points
Points	Energy (kcal)	Saturated Fat (g)	Total Sugars (g)	Sodium (mg)	Concentrated Fruits and Vegetables	Non-Concentrated fvnl	Protein (g)	Dietary Fiber (g)
	per 100 g or 100 mL	per 100 g or 100 mL	per 100 g or 100 mL	per 100 g or 100 mL	%	%	per 100 g or 100 mL	per 100 g or 100 mL
0	≤83	≤0.6	≤2.1	≤103	<25	< 40	≤3.0	≤0.7
1	>83	>0.6	>2.1	>103	≥25	≥40	>3.0	>0.7
2	>166	>1.2	>4.2	>206	≥43	≥60	>5.8	>1.4
3	>249	>1.8	>6.3	>309	≥52	≥67	>8.4	>2.1
4	>332	>2.4	>8.4	>412	≥63	≥75	>10.8	>2.8
5	>415	>3.0	>10.5	>515	≥67	≥80	>13.0	>3.5
6	>498	>3.6	>12.6	>618	≥80	≥90	>15.0	>4.3
7	>581	>4.2	>14.7	>721	≥90	≥95	>17.0	>5.2
8	>664	>4.8	>16.8	>824	=100	=100	>19.0	>6.1
9	>747	>5.4	>18.9	>927			>21.1	>7.1
10	>830	>6.0	>21.0	>1030			>23.6	>8.4
11	>913	>6.8	>24.6	>1133			>26.6	>9.8
12		>7.7	>28.2	>1236			>30.4	>11.6
13		>8.7	>32.2	>1339			>35.3	>13.8
14		>9.8	>36.6	>1442			>41.6	>16.6
15		>11.1	>40.8	>1545			>50.0	>20.0
16		>12.5	>45.7	>1648				
17		>14.2	>50.7	>1751				
18		>16.1	>55.7	>1854				
19		>18.4	>61.3	>1957				
20		>21.0	>67.1	>2060				
21		>24.1	>72.7	>2163				
22		>27.7	>79.1	>2266				
23		>31.9	>85.6	>2369				
24		>36.9	>92.0	>2472				
25		>42.8	>99.0	>2575				
26		>49.5		>2678				
27		>57.4		>2781				
28		>66.8		>2884				
29		>77.4		>2987				
30		>90.0		>3090				

**Table 4:** Distribution of food categories by HSR and NPM-PFJ (1.0).

	NPM-PFJ (1.0) Category						
HSR Category	1	2	3	4	5	6	Total
1. Beverages (non-dairy), including jellies and water-based ice confections	31		5		22		58
1D. Milk and Dairy beverages (and alternatives)	10						10
2. Foods	102	57	91	157	52	106	565
2D. Dairy foods (and alternatives)	5		19				24
3D. Cheese			11				11
Total	148	57	126	157	74	106	668

3. Oils and Spreads were not included.

**Table 5:** Numerical table of ratings for the NPM-PFJ (1.0).

Category	Description	Final Score	Rating	Category	Description	Final Score	Rating
1	Processed rice products Processed wheat and other grain products Processed corn products Soy milk Fish/mollusk/crustacean products (canned and paste products) Processed egg products Milk/dairy products and yogurt Beverages	≤−2	5	4	Noodles (dried) Vegetable products (pickles) Fish/mollusk/crustacean products (dried products and salted/simmered/pickled products)	≤−4	5
		−1–0	4.5			−3–−2	4.5
		1	4			−1–1	4
		2	3.5			2–4	3.5
		3	3			5–14	3
		4	2.5			15–16	2.5
		5	2			17–18	2
		NA	1.5			19–23	1.5
		6–7	1			24–29	1
		≥8	0.5			≥30	0.5
2	Soybean products (solid-form) Seed products	≤−16	5	5	Potato/sweet potato/other potato products Vegetable products (canned/frozen/100% juices) Fruits products (canned/frozen/100% juices) Mushroom products Algae products	≤−12	5
		−15–−13	4.5			−11	4.5
		−12–−8	4			−10–−9	4
		−7–−6	3.5			−8–−7	3.5
		−5	3			−6	3
		−4	2.5			−5–−4	2.5
		−3	2			−3–−2	2
		−2–−1	1.5			NA	1.5
		0–10	1			−1–0	1
		≥11	0.5			≥1	0.5
3	Meat products Cheeses, milk powders, ice creams, creams Western confectioneries and pastries	≤6	5	6	Fruits products (dried fruits, jams) Japanese confectioneries and candies	≤0	5
		7–10	4.5			1–5	4.5
		11–15	4			6–8	4
		16–17	3.5			9–11	3.5
		18–20	3			12–13	3
		21–22	2.5			14–15	2.5
		23–25	2			16–18	2
		26–29	1.5			19–20	1.5
		30–32	1			21–22	1
		≥33	0.5			≥23	0.5

Rating 5 = 0–10 percentile of the distribution of the final scores for each category. For each 0.5 rating decrease, the upper limit of final score is the +10th percentile value.

## Data Availability

All additional data are included in the Supplementary Materials.

## Supplementary Materials

The following supporting information can be downloaded at: https://www.mdpi.com/article/10.3390/nu16173026/s1, Table S1: Food composition data for 668 processed foods in Japan used in this study and their evaluation based on the HSR system and on the NPM-PFJ (1.0); Table S2: Basis for calculating the numerical tables of points for the NPM-PFJ (1.0); Table S3: Distribution of the final scores for the NPM-PFJ (1.0); Table S4: Basis for identifying non-condensed (%) fvnl.
